# Isolation of Isocoumarins and Flavonoids as α-Glucosidase Inhibitors from *Agrimonia pilosa* L.

**DOI:** 10.3390/molecules25112572

**Published:** 2020-05-31

**Authors:** Mi Jin Park, Young-Hwa Kang

**Affiliations:** Division of Applied Biosciences, College of Agriculture & Life Sciences Kyungpook National University, Daegu 41566, Korea; mj-7311@hanmail.net

**Keywords:** *Agrimonia pilosa* L., α-glucosidase inhibitory activity, isocoumarin, flavonoid, structure–activity relationship, industrial source

## Abstract

*Agrimonia pilosa* L. (AP) showed potent α-glucosidase inhibitory (AGI) activity, but it is uncertain what phytochemicals play a key factor. The phytochemical study of AP based on AGI activity led to the isolation of four isocoumarins; agrimonolide (**1**), agrimonolide-6-*O*-β-d-glucopyranoside (**2**), desmethylagrimonolide (**3**), desmethylagrimonolide-6-*O*-β-d-glucopyranoside (**4**), and four flavonoids; luteolin (**5**), quercetin (**6**), vitexin (**7**), and isovitexin (**8**). The four isocoumarins were isolated as α-glucosidase inhibitors for the first time. Isocoumarins, compound **1** (agrimonolide) and **3** (desmethylagrimonolide) showed strong α-glucosidase inhibitory activities with IC_50_ values of 24.2 and 37.4 µM, respectively. Meanwhile, isocoumarin and flavonoid glycosides showed weak AGI activity. In the kinetic analysis, isocoumarins, compounds **1** and **3** showed non-competitive inhibition, whereas flavonoid, compound **6** showed competitive inhibition.

## 1. Introduction

Diabetes is a common metabolic disease in modern society associated with the development of insulin resistance, peculiar glucose and lipid metabolism, impaired insulin signaling and β-cell dysfunction, sub-clinical inflammation, and increased oxidative stress [1]. It is classified into the following three main types: insulin-dependent diabetes (type 1), non-insulin-dependent diabetes (type 2, T2DM), and gestational diabetes. T2DM is the most common type of diabetes, and it has been reported to account for more than 90% of all cases of diabetes [2]. A therapeutic approach for treating T2DM in the early stage involves the decrease of postcibal hyperglycemia [3]. This is accomplished by delaying the absorption of glucose through the inhibition of carbohydrate digestive enzymes. Alpha-glucosidase [EC 3.2.1.20] is known to be a critical enzyme associated with T2DM in humans [3]. The α-glucosidase inhibitory (AGI) reaction is essential in T2DM patients owing to its potential effect in reducing glucose absorption through the prevention of carbohydrate digestion [3]. Inhibitors of α-glucosidase determine the reduction in the rate of postprandial glucose absorption and the rise in the levels of plasma glucose. Recent antidiabetic treatments involve synthetic drugs that very often have side effects [4]. In contrast, plant derived AGI agents have limited or no side effects owing to their metabolites [4]. Thus, plant-derived medicines and functional foods might be attractive alternatives to synthetic drugs for the prevention and treatment of T2DM [4]. Currently, many plant species are being used for the reduction of T2DM worldwide [5]. Recently, many studies have been conducted to search the active constituents of plants with antidiabetic potential. 

*Agrimonia pilosa* L. (AP) is a perennial plant from the Rosaceae family, which is distributed widely in the temperate regions of East Asia, including Korea. The aerial parts of AP have been used as a wild vegetable mainly in the mountain villages of Korea. The diverse biological activities of AP, including anticancer [6,7], antioxidant [8], and hepatoprotective activities [9] have been reported as being traditionally used for the treatment of T2DM [10]. The components of the flavonoid and triterpenoid fractions with the AGI activities from AP have been investigated indirectly using high-performance liquid chromatography (HPLC) analysis [10,11]. However, the bioactive compounds of AP with AGI activity have not been elucidated clearly. 

In the present study, the active principle of the antidiabetic property of AP was elucidated using the AGI activity-guided isolation method. The AGI action mechanisms of isolated active compounds were determined through enzyme kinetic studies, and the structure–activity relationship (SAR) was analyzed. 

## 2. Results and Discussion

The methanol extract from the aerial parts of *Agrimonia pilosa* L. (AP) showed potent α-glucosidase inhibitory (AGI) activity in our screening study. The hyperglycemic inhibitory property of the ethanol extract of AP has been reported previously by Liu and Na [10,11]. However, there is no report on the active fractions/compounds. Hence, the present study attempted to elucidate the hyperglycemic inhibitory property of AP extract. Successive activity-guided isolation of bioactive fractions was performed, and the AGI activities of the isolated active compounds were evaluated.

### 2.1. Isolation of Active Compounds from AP

Successive fractionation of the methanol extract of AP (APM) produced three fractions. Among them, the ethyl acetate fraction (APE) had the strongest AGI activity (IC_50_ of 18.8 µg/mL), and it was followed by the *n*-buthanol fraction (APB) (IC_50_ of 29.8 µg/mL) (Table 1). The hexane fraction (APH) had the lowest activity (IC_50_ of 60.7 µg/mL). The IC_50_ values represent the half-maximal AGI concentrations. All samples inhibited α-glucosidase activity in a dose-dependent manner. The subsequent bioactivity-guided fractionation of the APE fraction by open column chromatography led to the isolation of eight compounds (**1**–**8**). To our knowledge, this study is the first to report on α-glucosidase inhibitors isolated from AP through AGI-guided fractionation.

### 2.2. Structure Elucidation of Active Compounds from AP

A previous study investigated the AGI effects of fractions abundant in flavonoids and triterpenoids isolated from AP and found that these fractions had potent AGI effects [10]. Eight pure compounds were isolated from the active subfractions from the APE fraction. The structures of the compounds were identified using ^1^H- NMR, and ^13^C-NMR analyses, and their purities (>95%) were determined using HPLC. 

**Figure 1:**
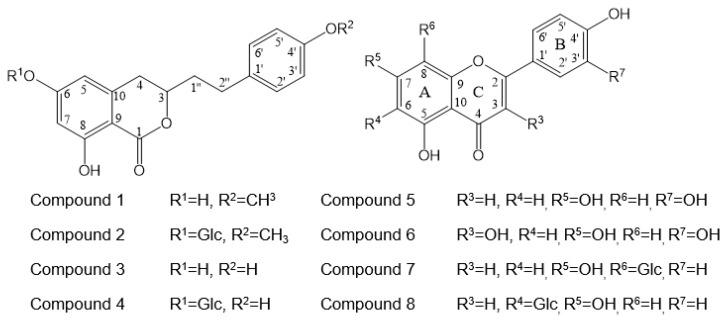
Chemical structure of active compounds **1**–**8** isolated from the *Agrimonia pilosa* L. (AP).

The active compounds were identified as agrimonolide (**1**) [9], agrimonolide-6-*O*-β-d-glucopyranoside (**2**) [9], desmethylagrimonolide (**3**) [9], desmethylagrimonolide-6-*O*-β-d-glucopyranoside (**4**) [12], luteolin (**5**) [13], quercetin (**6**) [13], vitexin (**7**) [14], and isovitexin (**8**) [8] through comparison of the spectral data with data from the literature. The structures of the compounds are shown in Figure 1. In this study, compound **3** was isolated for the first time from the methanol extract of the aerial parts of AP. The ^1^H and ^13^C NMR spectral data of compound **3** are represented in Table 2. The ^1^H spectrum of compound **3** showed resonances assignable to two meta coupled aromatic protons at *δ_H_* 6.18 (H-5) and *δ_H_* 6.48 (H-7), four A_2_X_2_-type aromatic ring protons at *δ_H_* 6.92 (H-2′, 6′) and *δ_H_* 6.77 (H-3′, 5′), and a methylene group at *δ_H_* 2.11 (H-1′′a), *δ_H_* 2.09 (H-1′′b), *δ_H_* 2.73 (H-2′′a), and *δ_H_* 2.50 (H-2′′b). 

### 2.3. AGI Activities of the Compounds Isolated from AP

**Figure 2:**
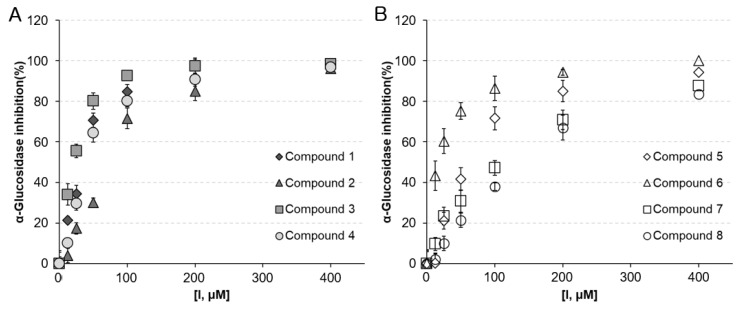
Dose-dependent effects of compounds **1**–**8** isolated from ethyl acetate fraction (APE) on the α-glucosidase inhibitory (AGI) activities. (**A**) % inhibition of isocoumarin compounds **1**–**4**, and (**B**) flavonoid compounds **5**–**8** against alpha-glucosidase.

Table 2 and Figure 2 shows the AGI activities of the active compounds **1**–**8** with IC_50_ values and inhibition percentages (%) at different concentrations. Compounds **1**–**8** exhibited significant dose-dependent AGI potential, with IC_50_ values of 24.2–117.6 μM. The Michaelis constant (K_m_ = 100 μM) was determined by plotting the initial rates normalized to the enzyme concentration (0.76 units/mL) versus the substrate concentration (50–200 μM). The AGI activities of compounds **3** (IC_50_ = 24.2 ± 0.6 μM, *K_I_* = 11.6 ± 0.4 μM), **6** (IC_50_ = 28.7 ± 1.2 μM, *K_I_* = 13.9 ± 0.8 μM), and **1** (IC_50_ = 37.4 ± 0.4 μM, *K_I_* = 17.3 ± 0.6 μM) were higher than that of acarbose (IC_50_ = 45.2 ± 1.2 μM, *K_I_* = 22.5 ± 01.2 μM), a representative α-glucosidase inhibitor used as positive control. Isocoumarin aglycone was more potent than its *O*-glycoside, as the isocoumarin aglycones **1** and **3** were more effective than the isocoumarin glycosides **2** (IC_50_ = 71.6 ± 0.4 μM, *K_I_* = 35.3 ± 1.2 μM) and **4** (IC_50_ = 52.3 ± 1.8 μM, *K_I_* = 26.2 ± 0.6 μM). The *O*-glucose moiety appears to reduce AGI activity. In general, *O*-glycosylation at any position on the aglycone reduces the enzyme inhibitory effect compared to that of aglycone [15]. Isocoumarin **3** (IC_50_ = 24.2 ± 0.6 μM, *K_I_* = 11.6 ± 0.4 μM) with a hydroxyl group at C-4′ was more potent than isocoumarin **1** (IC_50_ = 37.4 ± 0.4 μM, *K_I_* = 17.3 ± 0.6 μM) with a methoxy group (*δ*_c_ 55.83) at the same position. It means that the presence of hydroxyl groups on the C ring increases its AGI activity.

Among flavonoids, compound **6** (IC_50_ = 28.7 ± 1.2 μM, *K_I_* = 13.9 ± 0.8 μM), with hydroxyl substitution at the 3-position of the C-ring, was more effective than compounds **5** (IC_50_ = 65.8 ± 1.9 μM, *K_I_* = 33.1 ± 1.4 μM), **7** (IC_50_ = 117.6 ± 3.6 μM, *K_I_* = 57.6 ± 1.4 μM), and **8** (IC_50_ = 46.3 ± 1.7 μM, *K_I_* = 23.2 ± 0.8 μM). Compound **6** (quercetin) is one of the most common flavonoids in nature and is known to have excellent AGI activity owing to the presence of 3-OH groups, and the hydroxyl substitution on the C ring increases its AGI activity [16]. Meanwhile, the mechanisms of action along with in silico study findings and structure–activity relationships of flavonoids as α-glucosidase enzyme inhibitors indicate that free hydroxyl groups at C-3 are crucial [17]. Compound **7** (vitexin) showed the lowest AGI activity among flavonoids in AP. This finding indicates that the AGI potential reduces by glycosylation at the R6 position (A ring of C-8). 

### 2.4. AGI Kinetic Analysis of Active Isocoumarins 

**Figure 3:**
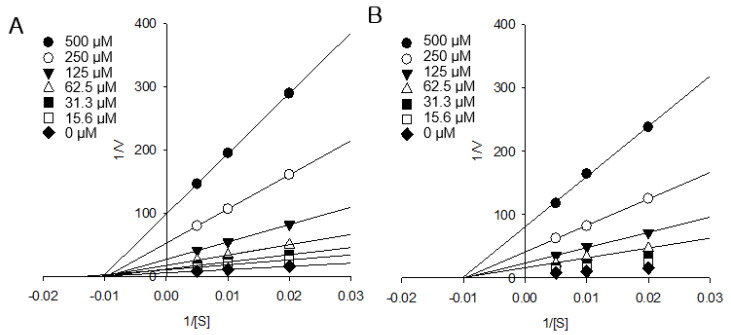
The kinetic models of compounds **1** and **3**. Lineweaver–Burk plot of (**A**) compound **1** and (**B**) compound **3** on the activity of alpha-glucosidase.

The AGI activity of isocoumarin compound **3** (IC_50_ = 2.4 ± 0.3 μM, *K_I_* = 1.2 ± 0.2 μM) was higher than that of flavonoid compound **6**, which is one of the most active α-glucosidase inhibitors (IC_50_ = 2.9 ± 0.6 μM, *K_I_* = 1.4 ± 0.3 μM). The excellent AGI effects and in silico study findings of flavonoids are well reported [18]. However, studies on the AGI effects of isocoumarins are rare. Recently, the AGI activity of isocoumarin was reported in fungus [19]. However, an AGI kinetic study of isocoumarins has not been reported. In the present study, isocoumarin compounds **1** and **3** exhibited strong AGI activity, and kinetic modes were carried out (Figure 3). In the AGI kinetic study, Lineweaver–Burk plots (1/V vs. 1/[S]) of isocoumarin compounds **1** and **3** showed that straight lines through different ranges of compound concentrations intersect in the second quadrant (Figure 3A,B). The straight lines of plots passing through the one point in a non-zero point on the x-axis are shown in Figure 3B. According to these kinetic data, compounds **1** and **3** showed non-competitive α-glucosidase inhibition. On the other hand, flavonoid compound **6**, which has excellent AGI activity, has been reported to show competitive α-glucosidase inhibition [20]. To our knowledge, this study is the first to present the α-glucosidase kinetics of isocoumarins. Our results are important because they indicate that not only flavonoids but also isocoumarins are good natural sources for α-glucosidase inhibition. Our comprehensive results suggest that a higher intake of AP by T2DM patients may provide some protection and treatment against the development of long-term complications of diabetes. 

## 3. Materials and Methods

### 3.1. AGI Activity Guided Fractionation and Isolation

**Figure 4:**
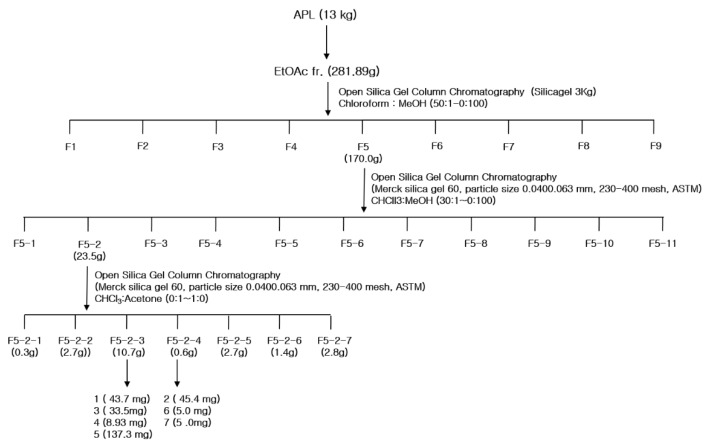
The fractionation scheme of *Agrimonia pilosa* L.

A total of 13 kg of AP (aerial parts) was washed with water, chopped, and shade dried, and its components were extracted with methanol using the Soxhlet extractor (Soxhlet Heater System WHM12295, DAIHAN Science, Won-Ju, Korea). After filtration, the extract was condensed under reduced pressure using a rotary evaporator, and the crude methanol extract (APM) was suspended in distilled water (8 L). The suspension was partitioned between hexane, ethyl acetate, and *n*-butanol. The extract was evaporated to dryness under reduced pressure to obtain the hexane fraction (APH), ethyl acetate fraction (APE), *n*-butanol fraction (APB), and the remaining aqueous fraction. The APE fraction (281.9 g), which was the most active fraction, was fractionated on an open column chromatography (Merck silica gel 60, particle size 0.0400.063 mm, 230–400 mesh, ASTM, Darmstadt, Germany) (Figure 4), and solvent systems involving CHCl_3_: MeOH (50:1, 30:1, 20:1, 10:1, 5:1, 3:1, 2:1, 1:1, and 0:100). Following thin layer chromatography (TLC) monitoring, the following nine major fractions were obtained: F1 (11.1 mg), F2 (0.1 mg), F3 (17.2 mg), F4 (56.8 mg), F5 (170.0 mg), F6 (8.6 mg), F7 (11.0 mg), F8 (6.4 mg), and F9 (0.1 mg). Among these fractions, F5 showed the strongest activity. F5 (170.0 g) was fractionated on a silica gel column using CHCl_3_ with an increasing portion of acetone, and 11 fractions (F5-1 to F5-11) were obtained. F5-2 (23.5 g) was further separated on a silica gel column using methylene chloride (DCM) with an increasing portion of MeOH. F5-2-3 and F5-2-4 fractions were purified with DCM:MeOH, and eight compounds were isolated. Compounds **1** (33.1 mg), **3** (43.7 mg), **5** (8.9 mg), and **6** (137.3 mg) were isolated from F5-2-3, and compounds **2** (45.4 mg), **4** (45.4 mg), **7** (5 mg), and **8** (5 mg) were isolated from F5-2-4. 

### 3.2. Agrimonolide *(**1**)*

Colorless powder. ^1^H NMR (500 MHz, CDCl_3_): *δ* 7.38 (d, *J* = 8.8 Hz, 2H, H-2′, 6′), 6.95 (d, *J* = 8.5 Hz, 2H, H-3′, 5′), 6.07 (d, *J* = 2.3 Hz, 1 H, H-7), 6.04 (d, *J* = 2.3 Hz, 1H, H-5), 5.38 (s, 1H, H-3), 3.82 (s, 3H, OCH_3_), 3.10 (ddd, *J* = 14.3, 9.4 and 5.3 Hz, 1H, H-2′′a), 2.79 (ddd, *J* = 14.0, 8.9 and 7.1 Hz, 1H, H-2′′b), 2.09 (m, 1H, H-1′′a), 1.97 (m, 1H, H-1′′b) (Figure S1). ^13^C NMR (125 MHz, CDCl_3_): *δ* 196.18 (C-1), 168.13 (C-8), 164.30 (C-6), 163.06 (C-4′), 160.21 (C-10), 130.55 (C-1′), 127.88 (C-2′, 6′), 114.39 (C-3′, 5′), 103.30 (C-5), 95.24 (C-7), 94.39 (C-9), 79.17 (C-3), 55.83 (OCH_3_), 55.53 (C-1′′), 43.36 (C-4), 29.85 (C-2′′) (Figure S2).

### 3.3. Agrimonolide-O-β-d-glucopyranoside *(**2**)*

Pale yellow powder. ^1^H NMR (500 MHz, DMSO-*d*_6_): *δ* 7.31 (d, *J* = 8.8 Hz, 2 H, H-2′, 6′), 6.92 (d, *J* = 8.5, 2H, H-3′, 5′), 6.47 (d, *J* = 2.3 Hz, 1 H, H-7), 6.17 (d, *J* = 2.3 Hz, 1 H, H-5), 5.38 (s, 1H, H-3), 3.84 (s, 3H, OCH_3_), 2.97 (ddd, *J* = 14.3, 9.4 and 5.3 Hz, 1H, H-2′′a), 2.50 (ddd, *J* = 14.0, 8.9 and 7.1 Hz, 1H, H-2′′b), 2.10 (m, 1H, H-1′′a), 1.92 (m, 1H, H-1′′b) (Figure S3). ^13^C NMR (125 MHz, DMSO- *d*_6_): *δ* 196.18 (C-1), 164.59 (C-8), 164.38 (C-6), 161.96 (C-4′), 157.77 (C-10), 146.21 (C-1′), 128.94 (C-2′, 6′), 113.84 (C-3′, 5′), 104.18 (C-5), 104.18 (C-9), 103.35 (C-7), 99.30 (glucopyranosyl C-1), 94.32 (glucopyranosyl C-5), 80.01 (glucopyranosyl C-3), 79.65 (glucopyranosyl C-2), 77.33 (glucopyranosyl C-4), 73.58 (glucopyranosyl C-6), 68.50 (OCH_3_), 36.81 (C-1′′), 33.19 (C-4), 30.67 (C-2′′) (Figure S4).

### 3.4. Desmethylagrimonolide *(**3**)*

Pale yellow powder. ^1^H NMR (500 MHz, DMSO- *d*_6_): *δ* 6.92 (d, *J* = 8.7 Hz, 2 H, H-2′, 6′), 6.77 (d, *J* = 8.5, 2H, H-3′, 5′), 6.48 (d, *J* = 2.3 Hz, 1 H, H-7), 6.18 (d, *J* = 2.3 Hz, 1 H, H-5), 4.38 (m, 1H, H-3), 2.73 (ddd, *J* = 14.3, 9.4 and 5.3 Hz, 1H, H-2′′a), 2.50 (ddd, *J* = 14.0, 8.9 and 7.1 Hz, 1H, H-2′′b), 2.11 (s, 1H, H-1′′a), 2.09 (s, 1H, H-1′′b) (Figure S5). ^13^C NMR (125 MHz, DMSO- *d*_6_): *δ* 169.94 (C-1), 163.77 (C-8), 163.72 (C-6), 158.18 (C-4′), 141.98 (C-10), 132.94 (C-1′), 129.02 (C-2′, 6′), 113.59 (C-3′, 5′), 106.91 (C-5), 102.14 (C-7), 78.68 (C-3), 36.40 (C-1′′), 32.56 (C-4), 29.72 (C-2′′) (Figure S6).

### 3.5. Desmethylagcimonolide-O-β-d-glucopyranoside *(**4**)*

Pale yellow powder. ^1^H NMR (500 MHz, DMSO- *d*_6_): *δ* 6.92 (d, *J*=8.7 Hz, 2 H, H-2′, 6′), 6.77 (d, *J* = 8.5 Hz, 2H, H-3′, 5′), 6.48 (d, *J* = 2.3 Hz, 1 H, H-7), 6.18 (d, *J* = 2.3 Hz, 1 H, H-5), 4.50 (m, 1H, H-3), 4.98–3.38 (m, 6H, glucopyranosyl H), 2.73 (ddd, *J* = 14.3, 9.4 and 5.3 Hz, 1H, H-2′′a), 2.50 (ddd, *J* = 14.0, 8.9 and 7.1 Hz, 1H, H-2′′b), 2.11 (s, 1H, H-1′′a), 2.09 (s, 1H, H-1′′b) (Figure S7). ^13^C NMR (125 MHz, DMSO-*d*_6_): *δ* 182.6 (C-1), 164.4 (C-6), 163.0 (C-8), 161.6 (C-4′), 160.3 (C-10), 156.5 (C-1′), 129.5 (C-2′,6′), 116.3 (C-3′, 5′), 110.0 (C-5), 107.6 (C-9), 101.8 (C-7), 99.8 (glucopyranosyl C-1), 78.4 (glucopyranosyl C-5), 78.0 (glucopyranosyl C-3), 74.8 (glucopyranosyl C-2), 71.3 (glucopyranosyl C-4), 62.5 (glucopyranosyl C-6), 37.9 (C-1′′), 31.2 (C-2′′) (Figure S8).

### 3.6. Luteolin *(**5**)*

Yellow powder. ESI-MS: ^1^H NMR (500 MHz, DMSO-*d*_6_): *δ* 12.99 (s, 1 H, 5-OH), 7.42 (m, 2 H, H2′, 6′), 6.90 (d, *J* = 8.0 Hz, 1 H, H-5′), 6.68 (s, 1 H, H-3), 6.46 (d, *J* = 2.0 Hz, 1 H, H-8), 6.20 (d, *J* = 2.0 Hz, 1 H, H-6) (Figure S9). ^13^C NMR (125 MHz, DMSO-*d*_6_): *δ* 182.13 (C-4), 164.59 (C-7), 164.37 (C-2), 161.96 (C-5), 157.77 (C-9), 150.17 (C-4′), 146.21 (C-3′), 121.99 (C-1′), 119.47 (C-6′), 116.49 (C-5′), 113.84 (C-2′), 104.18 (C-10), 103.36 (C-3), 99.30 (C-6), 94.32 (C-8) (Figure S10). 

### 3.7. Quercetin *(**6**)*

Yellow powder. ^1^H NMR (500 MHz, DMSO-*d*_6_): *δ* 12.50 (s, 1 H, 5-OH), 10.78 (s, 1 H, H-7), 9.60 (s, 1 H, H-4′), 9.37 (s, 1 H, H-3), 9.31 (s, 1 H, H-3′), 7.69 (d, *J* = 2.18 Hz, 1 H, H-4′), 7.54 (dd, *J* = 2.23 and 6.28 Hz, 1H, H-6′), 6.90 (d, *J* = 8.5 Hz, 1 H, H-5′), 6.42 (d, *J* = 2.0 Hz, 1 H, H-8), 6.20 (d, *J* = 2.0 Hz, 1 H, H-6) (Figure S11). ^13^C NMR (125 MHz, DMSO-*d*_6_): *δ* 176.31 (C-4), 164.4 (C-7), 161.19 (C-5), 156.61 (C-9), 148.17 (C-4′), 147.28 (C-2), 145.53 (C-3′), 136.20 (C-3), 122.43 (C-1′), 120.45 (C-6′), 116.08 (C-5′), 115.54 (C-2′), 103.49 (C-10), 98.65(C-6), 93.82 (C-8) (Figure S12).

### 3.8. Vitexin *(**7**)*

Yellow powder. ^1^H NMR (500 MHz, DMSO-*d*_6_): *δ* 13.18 (s, 1H, 5-OH), 8.04 (d, 2H, *J* = 8.7 Hz, H-2′, 6′), 6.90 (d, 2H, *J* = 8.7 Hz, H-3′, 5′), 6.79 (s, 1H, H-3), 6.29 (s,1H, H-6), 4.70 (d, 1H, *J* = 9.8 Hz, glucopyranosyl H-1′), 4.06–3.22 (m, 6H, glucopyranosyl H) (Figure S13). ^13^C NMR (125 MHz, DMSO-*d*_6_): *δ* 182.59 (C-4), 164.44 (C-2), 163.05 (C-7) 161.62 (C-4′), 160.88 (C-5),156.48 (C-9), 129.45 (C-2′, 6′), 121.10 (C-1′), 116.30 (C-3′, 5′), 105.10 (C-10), 104.53 (C-8),102.93 (C-3), 98.62 (C-6), 82.33 (glucopyranosyl C-5), 79.15 (glucopyranosyl C-3), 73.83 (glucopyranosyl C-1), 71.32 (glucopyranosyl C-2), 71.03 (glucopyranosyl C-4), 61.78 (glucopyranosyl C-6) (Figure S14).

### 3.9. Isovitexin *(**8**)*

Yellow powder. ^1^H NMR (500 MHz, DMSO-*d*_6_): *δ* 13.57 (s, 1H, OH-5), 7.94 (d, 2H, *J* = 8.8 Hz, H-2′, 6′), 6.94 (d, 2H, *J* = 8.8 Hz, H-3′, 5′), 6.80 (s, 1H, H-3), 6.53 (s, 1H, H-8), 4.61 (d, 1H, *J* = 9.8 Hz, glucopyranosyl H-1), 4.07–3.13 (m, 6H, glucopyranosyl H) (Figure S15). ^13^C NMR (125 MHz, DMSO-*d*_6_) δ: 182.45 (C-4), 164.01 (C-2), 163.76 (C-7), 161.66 (C-4′), 160.61 (C-5), 156.70 (C-9), 128.96 (C-2′, 6′), 121.60 (C-1′), 116.47 (C-3′, 5′), 109.37 (C-6), 103.90 (C-10), 103.28 (C-3), 94.11 (C-8), 82.06 (glucopyranosyl C-5), 79.42 (glucopyranosyl C-3), 73.54 (glucopyranosyl C-1), 71.09 (glucopyranosyl C-2), 70.70 (glucopyranosyl C-4), 61.96 (glucopyranosyl C-6) (Figure S16).

### 3.10. Alpha-Glucosidase Inhibition (AGI) Assay

The preparation of the AP extract is presented in Section 2.1. The AGI assay [21] was performed with some modifications. The Michaelis constant (K_m_ = 100 μM) was determined by plotting the initial rates normalized to enzyme concentration (0.76 U mL^−1^) versus substrate concentration (50, 100, and 200 μM), and was compared with acarbose as a positive control. Briefly, 50 μL of 4 mmol *p*-nitrophenyl-*α*-d-glucopyranoside in buffer, as a substrate (1[S] = 100 μM), was pre-mixed with 100 μL of the sample (15.6–500 μM) in 100 mmol-phosphate buffer (pH 6.9), in a 96 well plate and incubated at 25 °C for 3 min. Then, 47–50μL of α-glucosidase (0.76 unit/mL in 100 mmol-phosphate buffer (pH 6.9), keep on the ice) was added and we set the final volume to 200 μL with 100 mmol-phosphate buffer (pH 6.9) and mixed well, and absorbance was measured in kinetic mode at 405 nm for 5 min with a spectrophotometer. The AGI activity the samples were calculated using the equation:

% inhibition = [1 − (A_c_ − A_s_/A_c_ − A_b_)] × 100 (1)

where A_c_ represents the initial absorbance, A_s_ represents the absorbance in the presence of the sample and A_b_ represents the absorbance in the absence of sample after the reaction. Acarbose, represents the inhibitor, was used as a positive control.

The inhibition constant (*K_I_*) values were calculated using the following equation:

*K_I_* = IC_50_/[1 + ([S]/K_m_)] (2)

where, IC_50_ was the half-maximal inhibitory concentration, [S] was the substrate concentration, and K_m_ was the substrate concentration at the half of V_max_. 

### 3.11. AGI Kinetic Analysis

Four isocoumarins **1**–**4** and four flavonoids **5**–**8** were identified from the APE fraction, and AGI kinetic analyses were performed. During the reaction, α-glucosidase (0.76 unit/mL in 100-mmol phosphate buffer [pH 6.9]) activity was inhibited by the compounds (I) in the presence of the substrate (4-mmol *p*-nitrophenyl-α-d-glucopyranoside in 100-mmol phosphate buffer [pH 6.9]). The inhibition modes (competitive, uncompetitive, non-competitive, or mixed) of isocoumarins **1**–**4** and flavonoids **5**–**8** were examined using nonlinear regression Michaelis–Menten enzyme kinetics. The Lineweaver–Burk plot methods were used to determine kinetic parameters at increasing substrate and compound concentrations, and the parameters were calculated using a nonlinear regression program (Sigma Plot; SPCC Inc., Chicago, IL, USA). The obtained results represent three independent experiments.

### 3.12. HPLC Analysis

The purity of the obtained compounds was analyzed using high performance liquid chromatography with photodiode array detector (HPLC-PDA, Shimadzu, Kyoto, Japan) analysis. Sample aliquots were filtered through a 0.2 µm membrane filter. The separations were performed on a YMC Triart C_18_ column (250 × 4.6 mm, 5 μm) maintained at 40 °C. The separation solvent A and B comprised of water and methanol with 0.5% H_3_PO_4_. The gradient solvent system was programmed: 0 min, 15% B; 0–5 min, 15–30% B; 5–12 min, 30–40% B; 12–18 min, 40–50% B; 18–25 min, 50–55% B; 25–35 min, 55–100% B; wash to 38 min with 100% B; and a 5 min recycle time. The flow rate was 1 mL/min. The peak detection was observed with a PDA detector at 200–800 nm.

### 3.13. NMR Analysis

Structure elucidation of the eight compounds from the APE was carried out by spectral techniques [^1^H- NMR, ^13^C-NMR and mass spectrometry]. The ^1^H NMR and ^13^C NMR spectra were recorded on a Bruker Avance Ⅲ 500 and the chemical shift was referenced to the solvent peak (*d_H_* 2.5 and *d_C_* 39.5 in DMSO-*d*_6_).

### 3.14. Statistical Analysis

Data are presented as mean ± standard deviation (triplicate experiments) unless otherwise indicated. Statistical analyses were performed using the general linear model procedure (GLM) in the SAS statistical software (Version 9.0, SAS Institute, Cary, NC, USA). Differences at *p* < 0.01 were considered statistically significant.

## 4. Conclusions

Eight compounds with AGI potential were identified from AP, and their AGI activities were successfully determined. Our results suggest that the glucose-lowering effect of AP is attributable to the strong AGI activities of compounds **1**, **3**, and **6**. With regard to α-glucosidase inhibition, isocoumarin compounds **1** and **3** show non-competitive inhibition, whereas flavonoid compound 6 shows competitive inhibition. Moreover, we found that aglycones of isocoumarin and flavonoid showed more powerful AGI activities than their glycosides. Additionally, the desmethylagrimonolide with a hydroxyl moiety at the C-4′ exhibited strong AGI activity compared to agrimonolide with a methoxy group at the C-4′. This result suggested that AGI activity was increased when the hydroxyl groups attached on the C-4′.

In the present study, although the enzyme inhibitory activities of the isolated compounds were analyzed in vitro, we believe that the results are relevant to the action mechanisms of hyperglycemia prevention in vivo. Our findings provide scientific support for the use of AP extract in traditional medicine for the prevention and treatment of T2DM. AP and its active compounds might be helpful in the development of medications and functional foods for T2DM.

## Figures and Tables

**Table 1:** α-Glucosidase inhibitory activities of the extracts and the fractions from *Agrimonia pilosa* L.

Extract/Fraction	IC_50_ (µg/mL)	Fr.	IC_50_ (µg/mL)	Sub-Fr.	IC_50_ (µg/mL)	Sub-Fr.	IC_50_ (µg/mL)
Methanol	47.7 ± 3.2	F1	330 ± 0.2	F5-1	15.5 ± 1.6	F5-2-1	51.0 ± 6.1
Hexane	60.7 ± 4.1	F2	62.3 ± 2.4	F5-2	4.0 ± 2.6	F5-2-2	28.5 ± 2.6
Ethyl acetate	18.8 ± 2.5	F3	51.3 ± 3.3	F5-3	46.3 ± 3.6	F5-2-3	7.5 ± 3.6
*n*-Buthanol	29.8 ± 2.7	F4	23.3 ± 0.9	F5-4	>100	F5-2-4	13.3 ± 4.6
		F5	19.2 ± 8.4	F5-5	>100	F5-2-5	>100
		F6	34.9 ± 6.1	F5-6	87.5 ± 6.6	F5-2-6	>100
		F7	70.0 ± 1.8	F5-7	96.9 ± 7.6	F5-2-7	>100
		F8	67.0 ± 6.4	F5-8	>100	-	-
		F9	70.5 ± 2.6	F5-9	>100	-	-
		-	-	F5-10	97.1 ± 10.6	-	-
		-	-	F5-11	61.3 ± 6.4	-	-

All extracts were examined in a set of experiments repeated three times. IC_50_ values of compounds represent the concentration that caused 50% enzyme activity loss.

**Table 2:** AGI activity of compounds **1**–**8** from *Agrimonia pilosa* L. with IC_50_ (µM) values.

Number/Compound.	IC_50_ (µM) ^a^	Inhibition Mode	*K_I_* (µM) ^b^
1	37.4 ± 0.4	Non-competitive	17.3 ± 0.6
2	71.6 ± 0.4	NT ^c^	35.3 ± 1.2
3	24.2 ± 0.6	Non-competitive	11.6 ± 0.4
4	52.3 ± 1.8	NT	26.2 ± 0.6
5	65.8 ± 1.9	NT,	33.1 ± 1.4
6	28.7 ± 1.2	Competitive [19]	13.9 ± 0.8
7	117.6 ± 3.6	NT	57.6 ± 1.4
8	46.3 ± 1.7	NT	23.2 ± 0.8
Acarbose ^d^	45.2 ± 1.2	Competitive [19]	22.5 ± 1.2

All compounds were examined in a set of experiments repeated three times. ^a^ IC_50_ values of compounds represent the concentration that caused 50% enzyme activity loss. ^b^ Values of inhibition constant. ^c^ NT is not tested. ^d^ Positive control.

## Supplementary Materials

The ^1^H NMR and ^13^C NMR spectrum data of compounds **1**–**8** are detailed in the Supplementary Materials (Figures S1–S16).
