# Supplementary material for: Isolation of Isocoumarins and Flavonoids as α-Glucosidase Inhibitors from Agrimonia pilosa L
Source: Molecules. 2020 May 31;25(11):2572. doi: 10.3390/molecules25112572 (PMC7321404; doi:10.3390/molecules25112572)
Supplement: Supplementary file 1 [file molecules-25-02572-s001.pdf]

Supplementary Materials

Compound 1 (Agrimonolide)

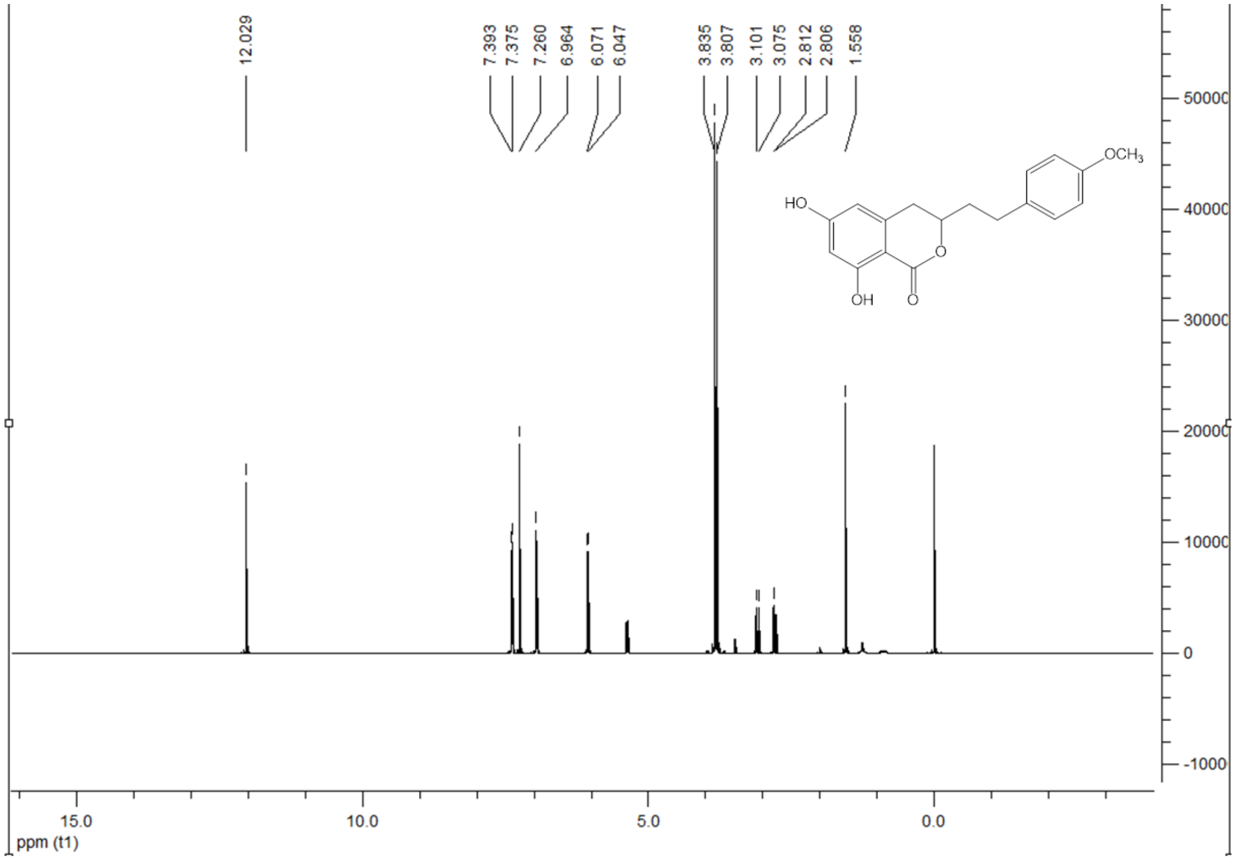

Figure S1. ^{1}H NMR spectrum of compound **1**.

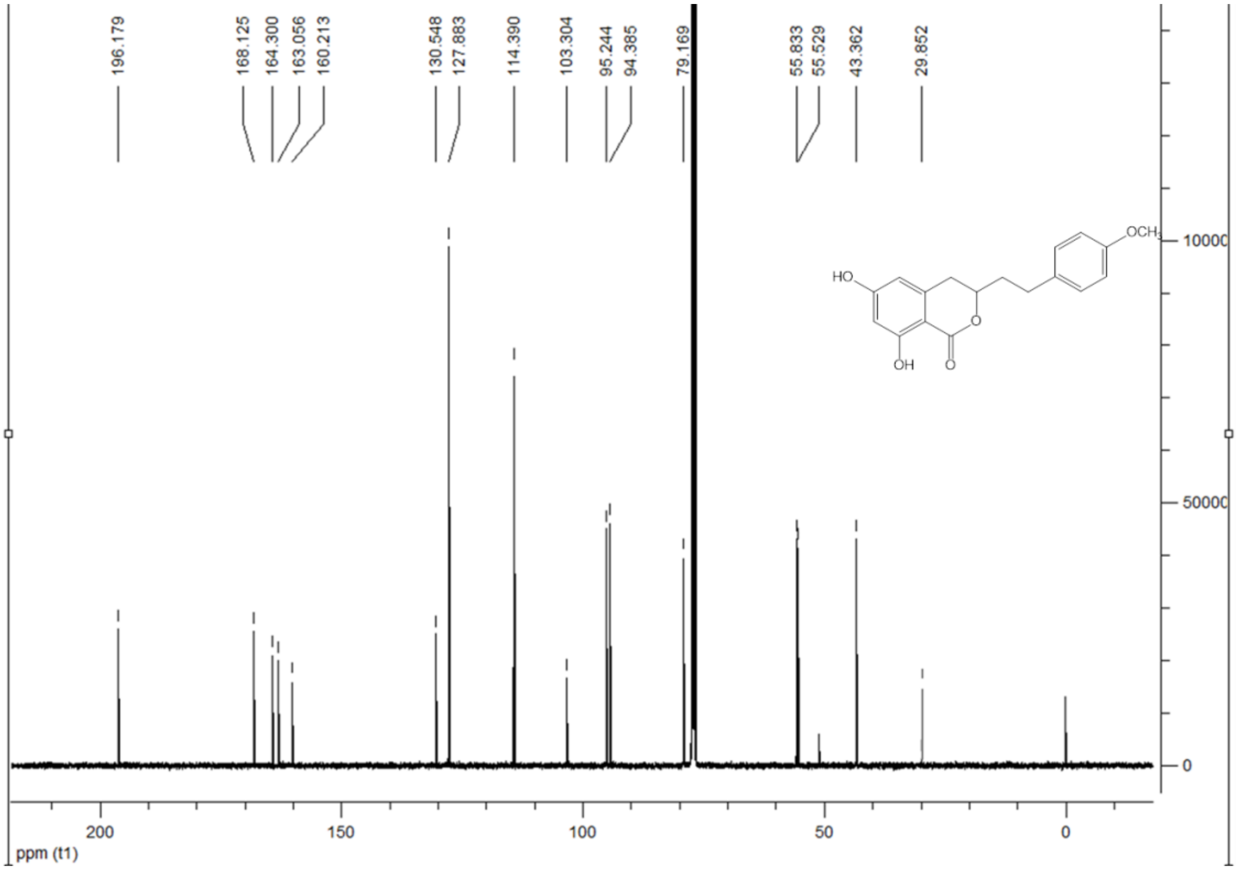

Figure S2. ^{13}C NMR spectrum of compound **1**.

Compound 2 (Agrimonolide-*O*-β-D-glucopyranoside)

.

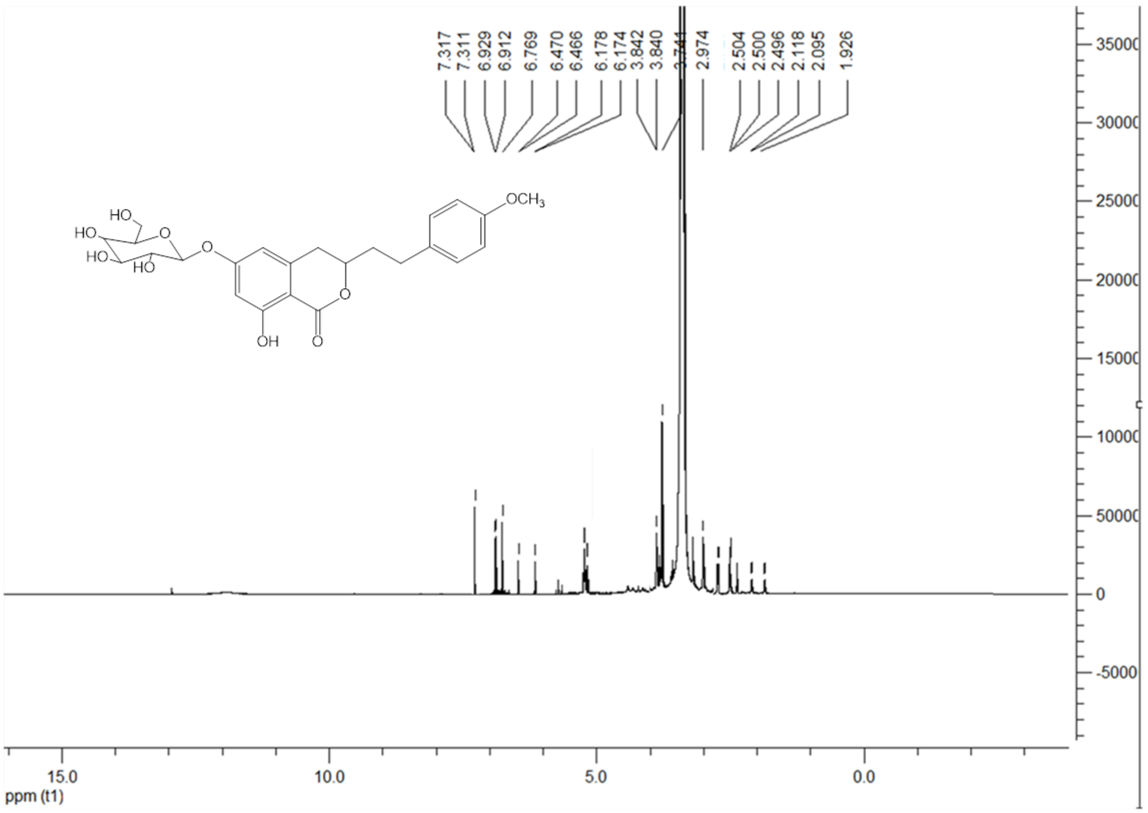

Figure S3. ^{1}H NMR spectrum of compound **2**.

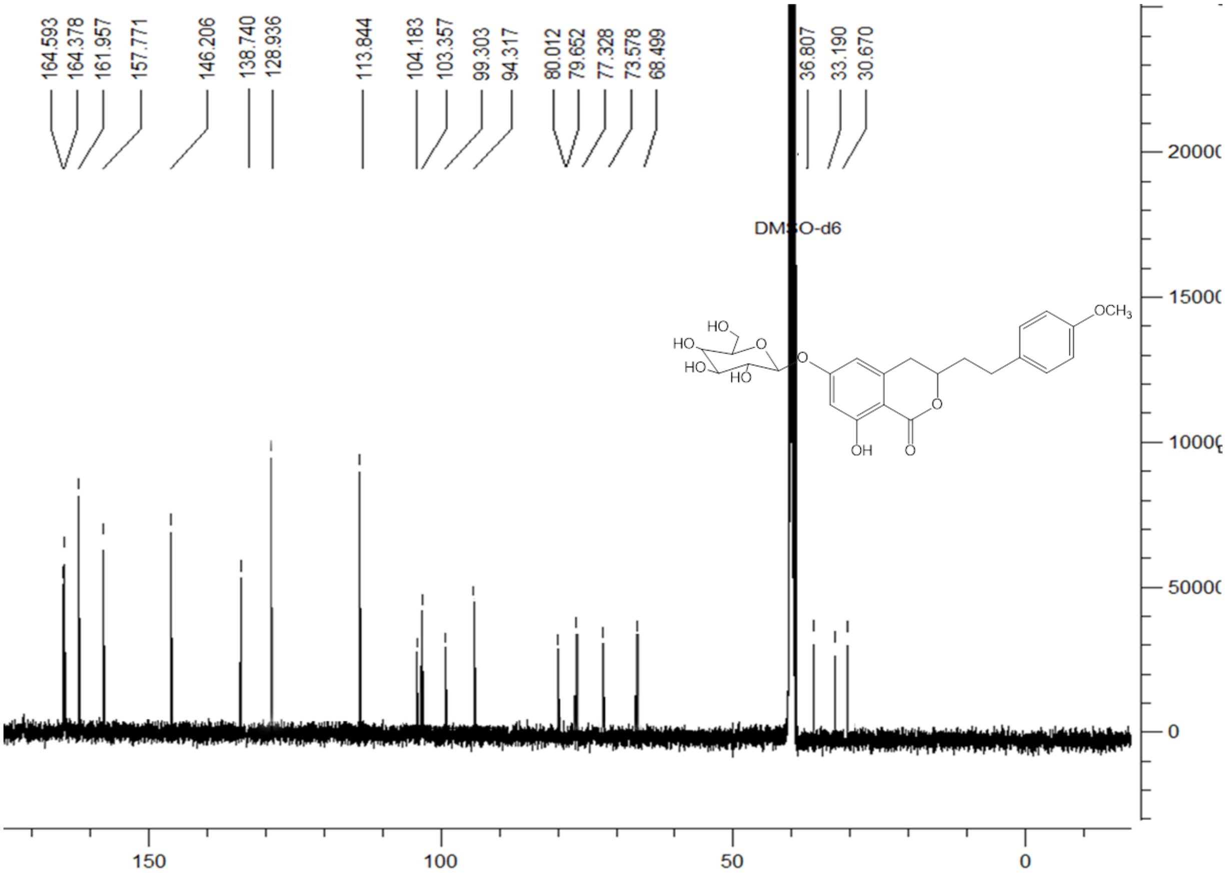

Figure S4. ^{13}C NMR spectrum of compound **2**.

Compound 3 (Desmethylagrimonolide)

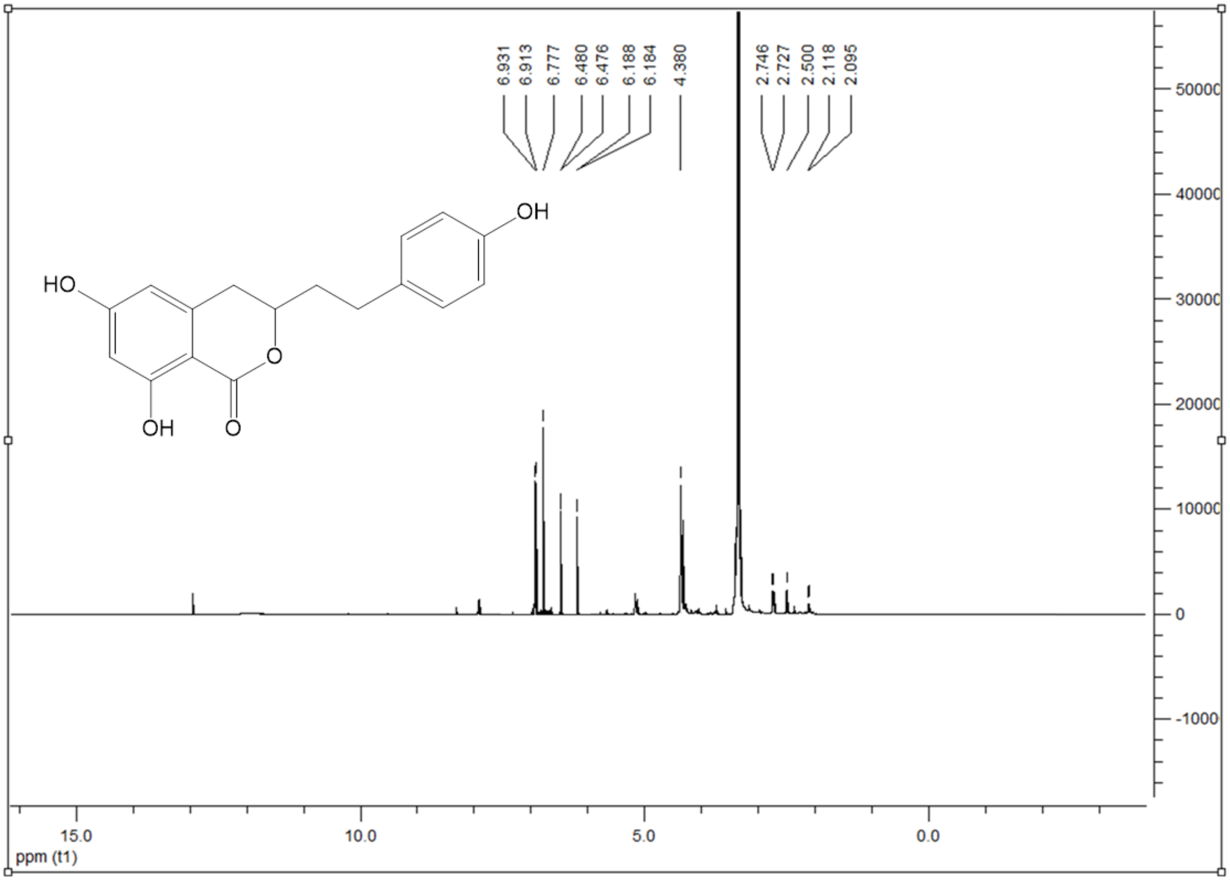

Figure S5. ^{1}H NMR spectrum of compound **3**.

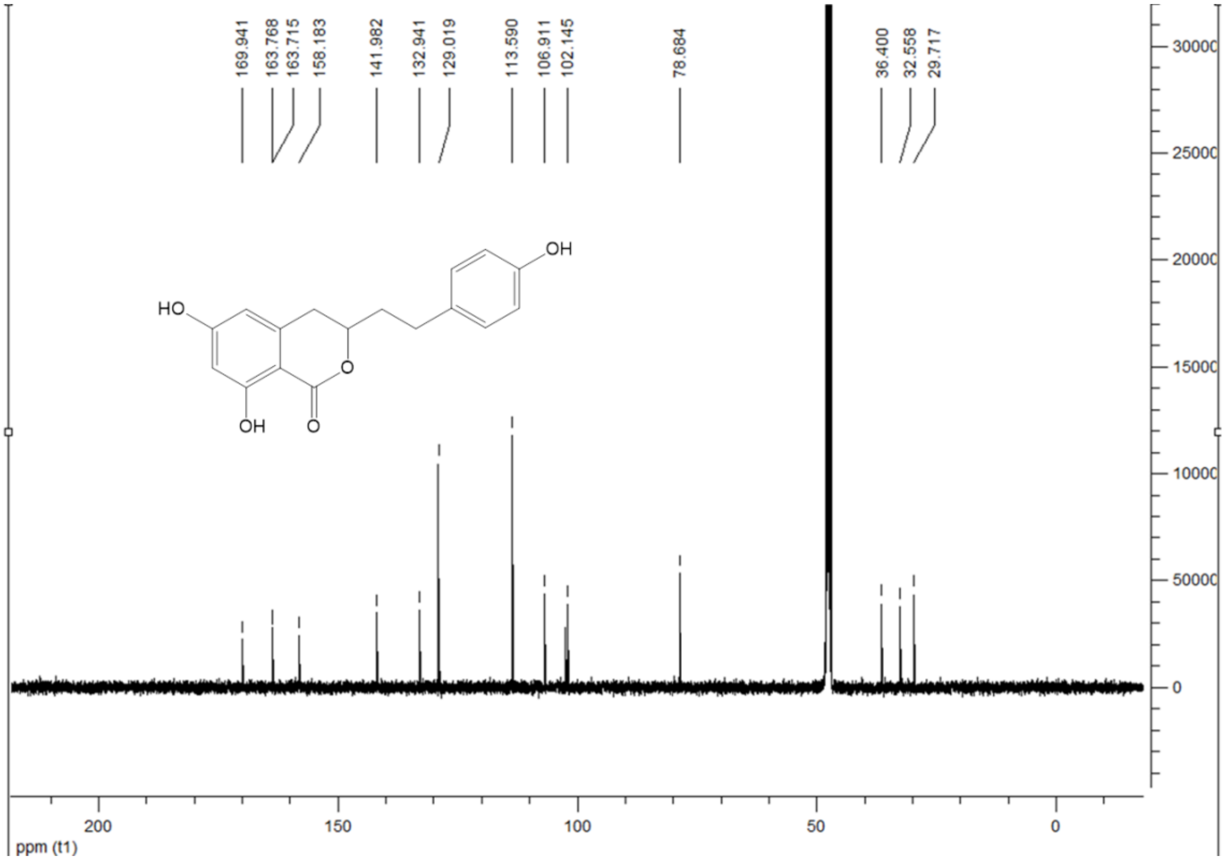

Figure S6. ^{13}C NMR spectrum of compound **3**.

Compound 4 (Desmethylagcimonolide-*O*-β-D-glucopyranoside)

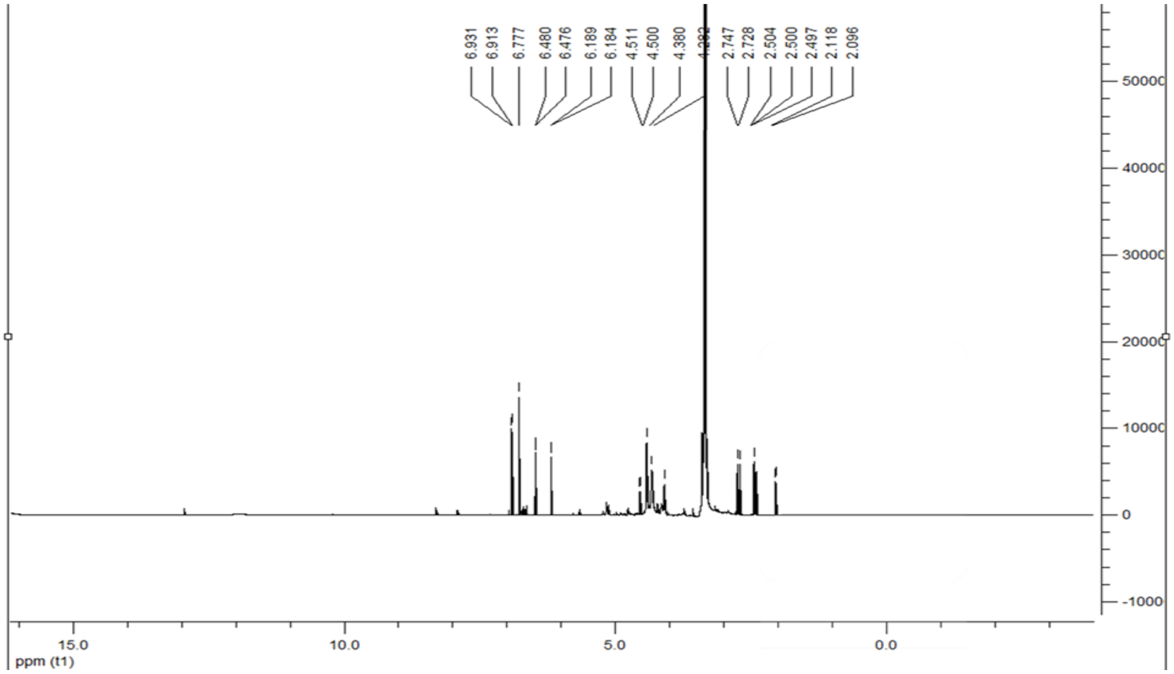

Figure S7. ^{1}H NMR spectrum of compound **4**.

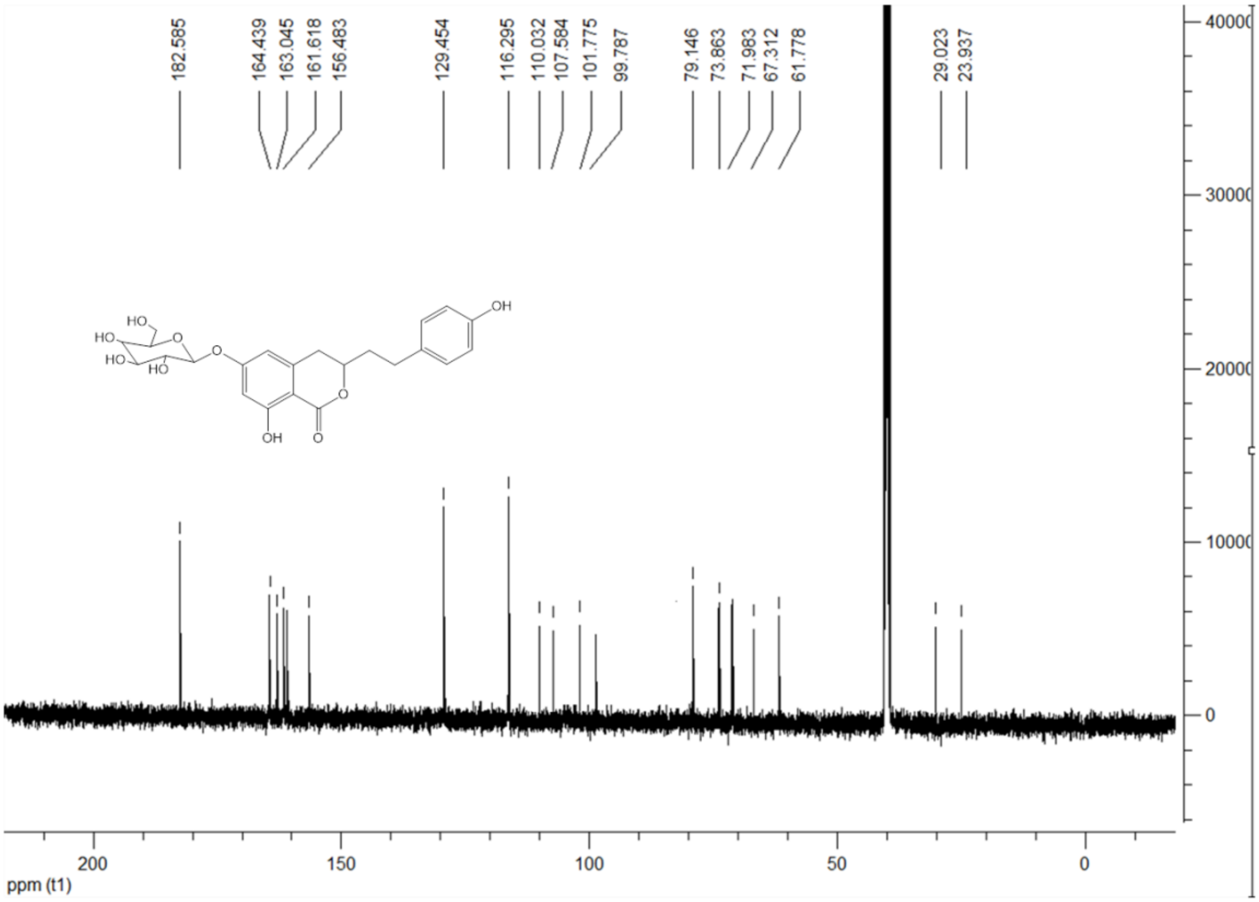

Figure S8. ^{13}C NMR spectrum of compound **4**.

Compound 5 (Luteolin)

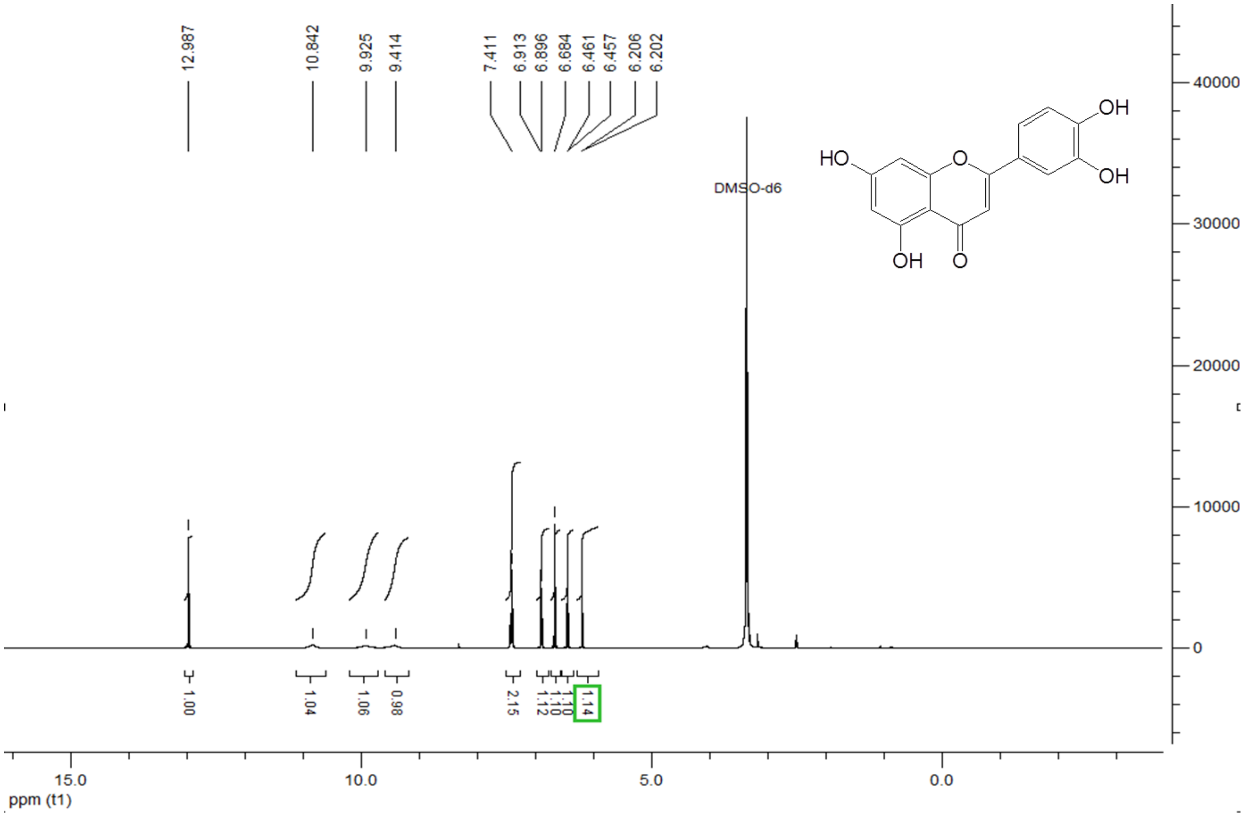

Figure S9. ^{1}H NMR spectrum of compound **5**.

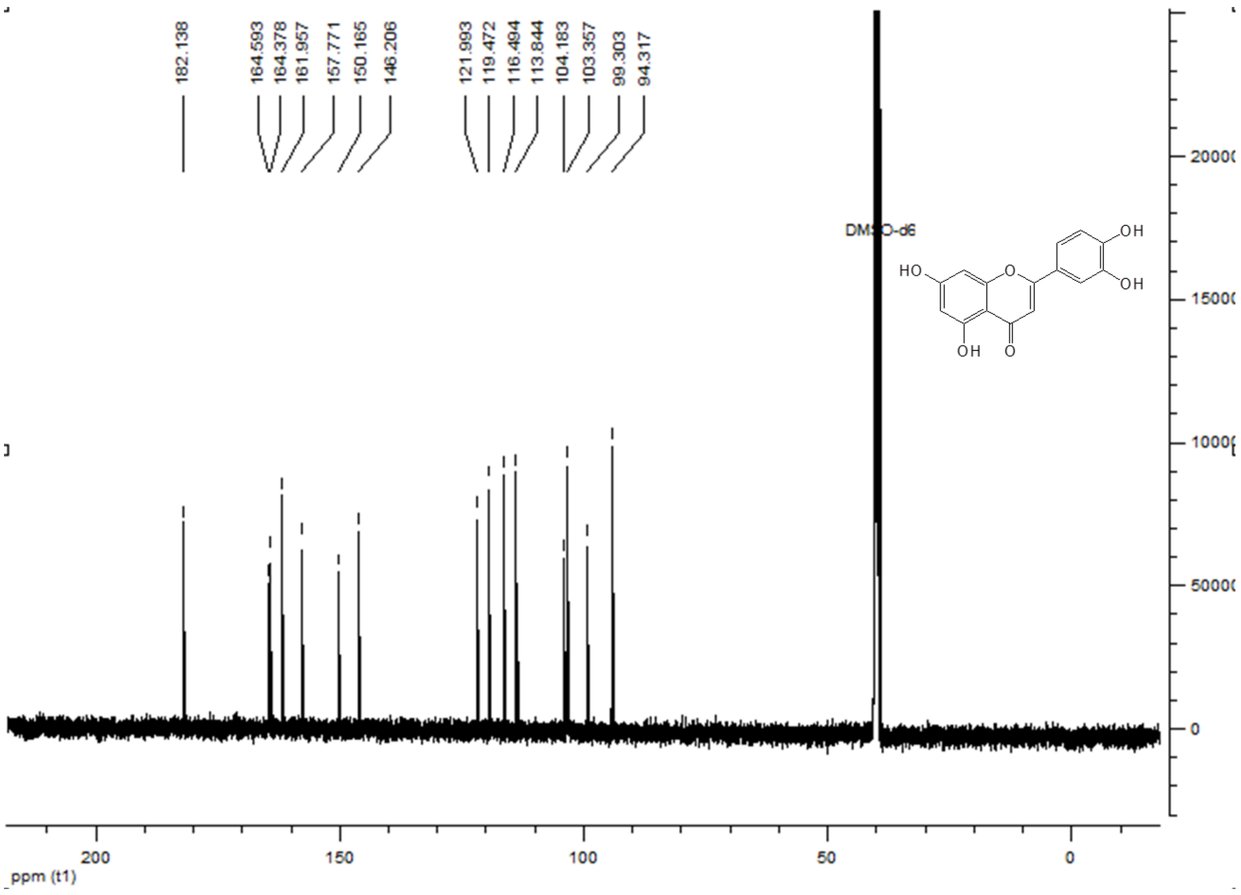

Figure S10. ^{13}C NMR spectrum of compound **5**.

Compound 6 (Quercetin)

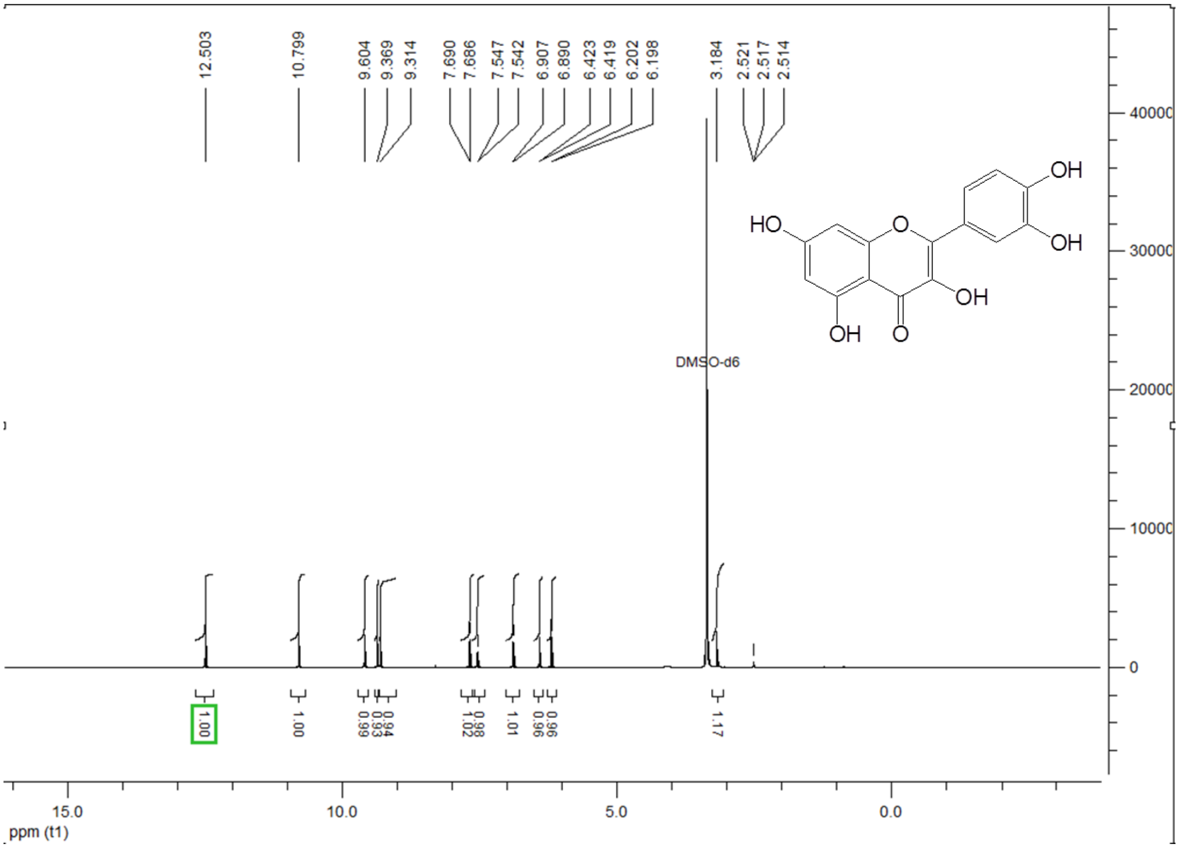

Figure S11. ^{1}H NMR spectrum of compound **6**.

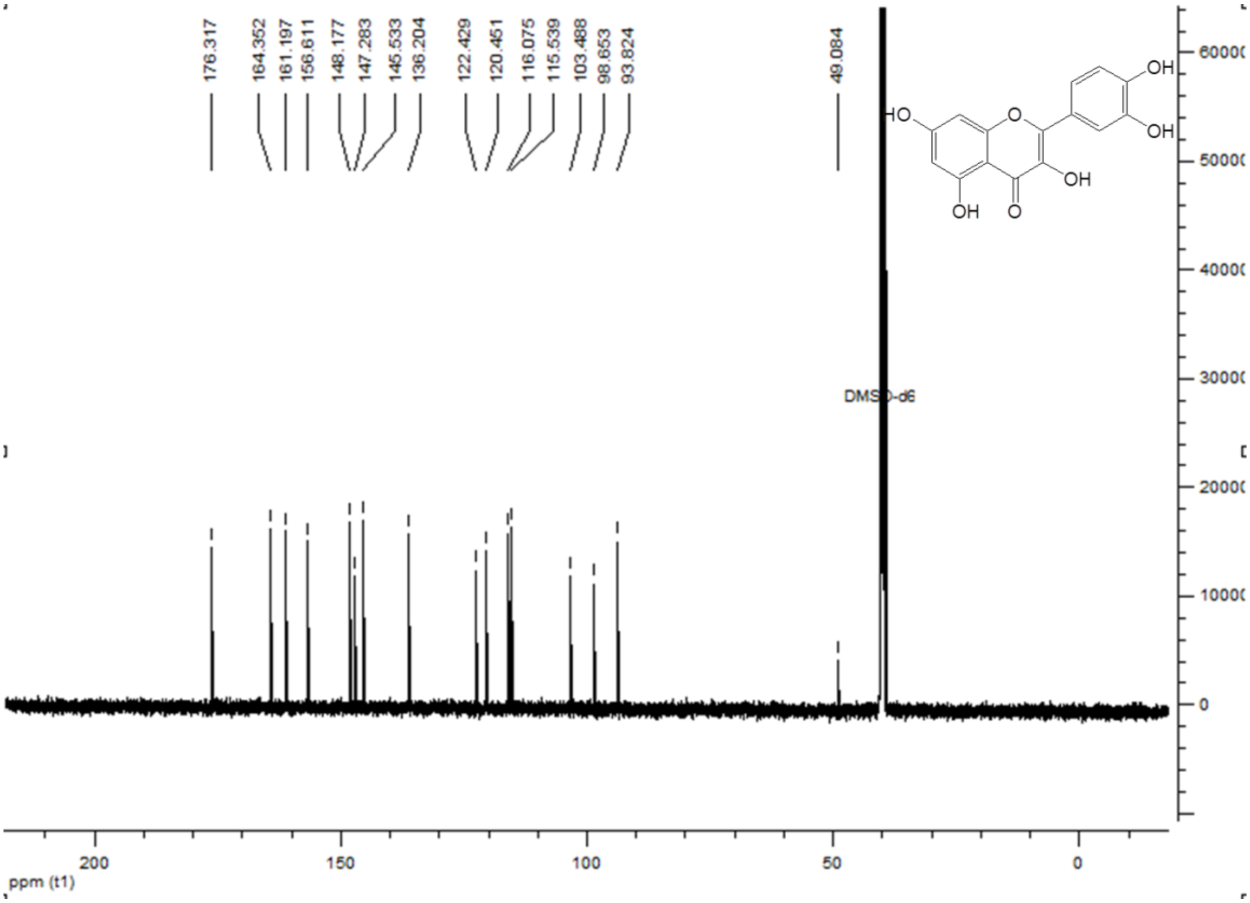

Figure S12. ^{13}C NMR spectrum of compound **6**.

Compound 7 (Vitexin)

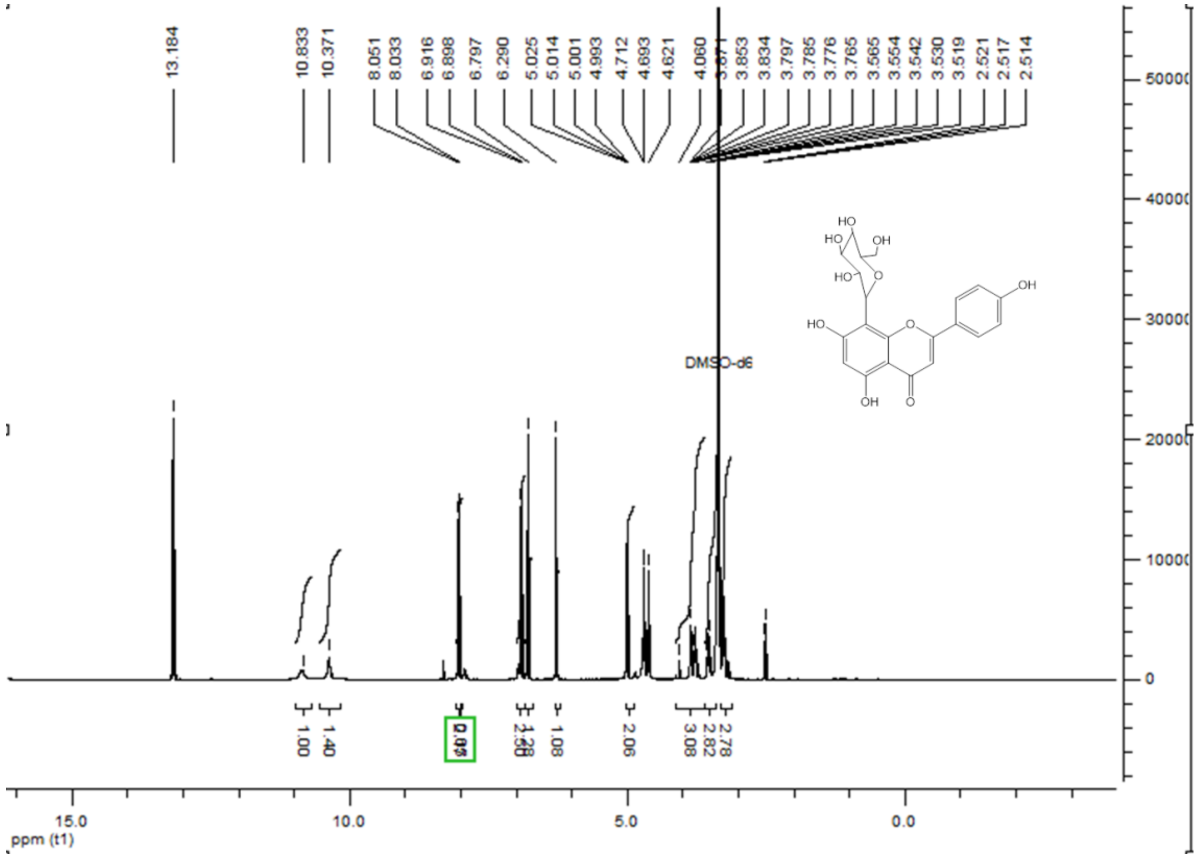

Figure S13. ^{1}H NMR spectrum of compound **7**.

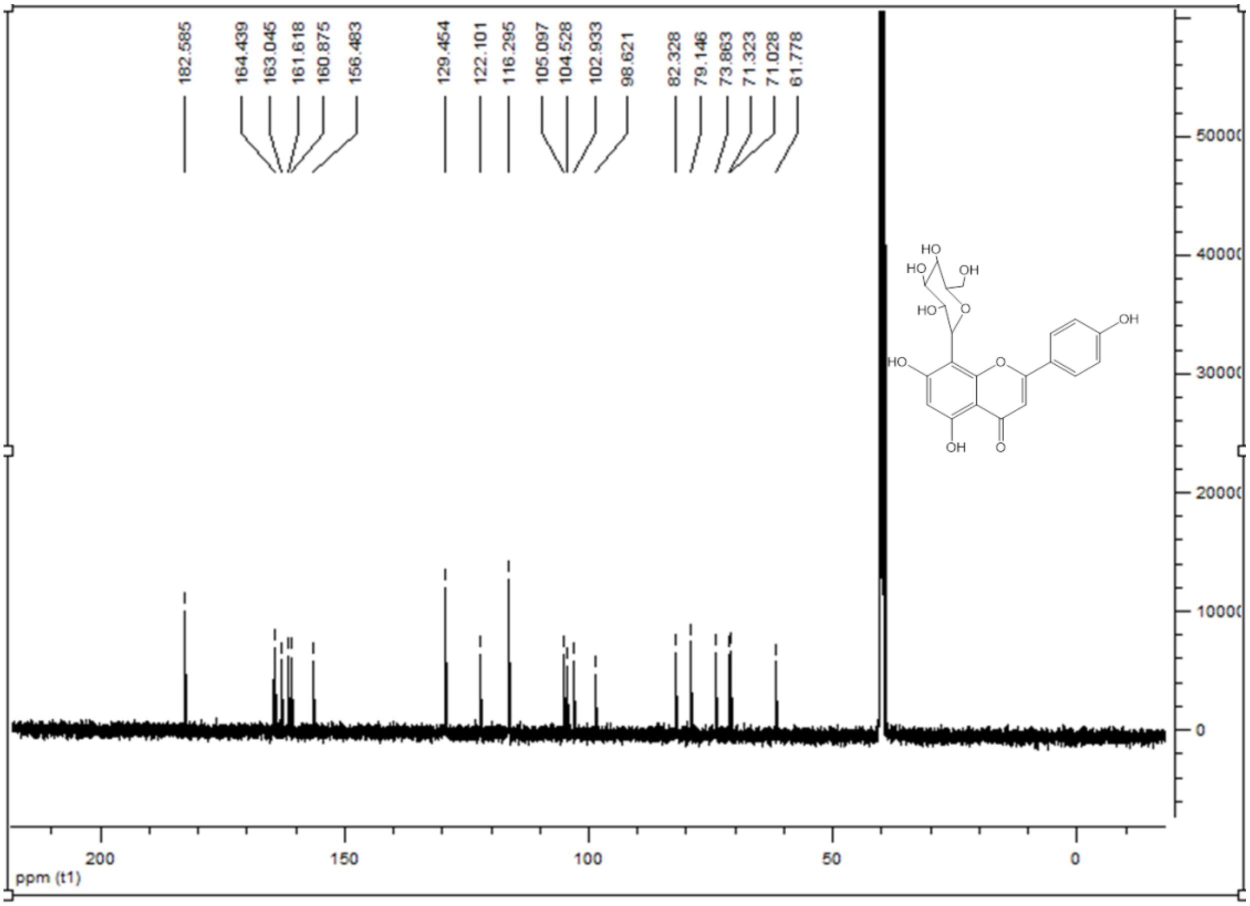

Figure S14. ^{13}C NMR spectrum of compound **7**.

Compound 8 (Isovitexin)

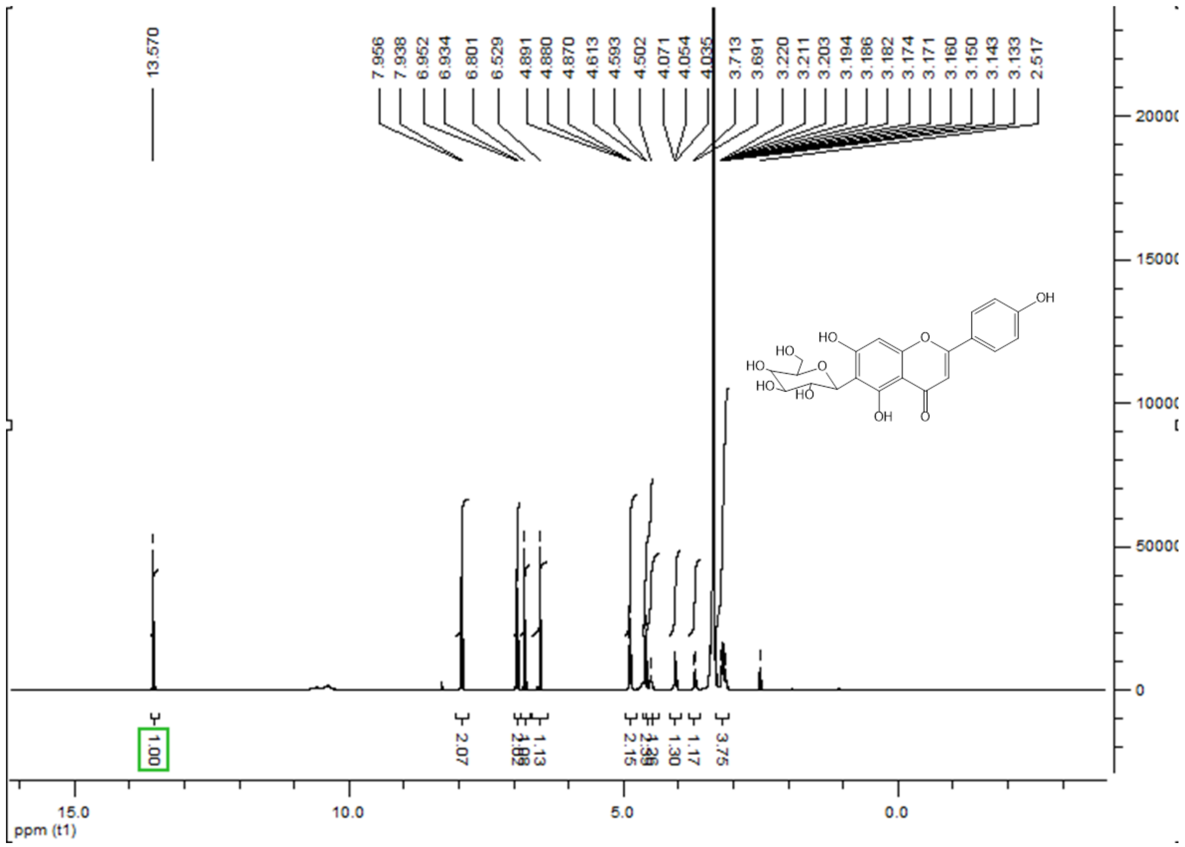

Figure S15. ^{1}H NMR spectrum of compound **8**.

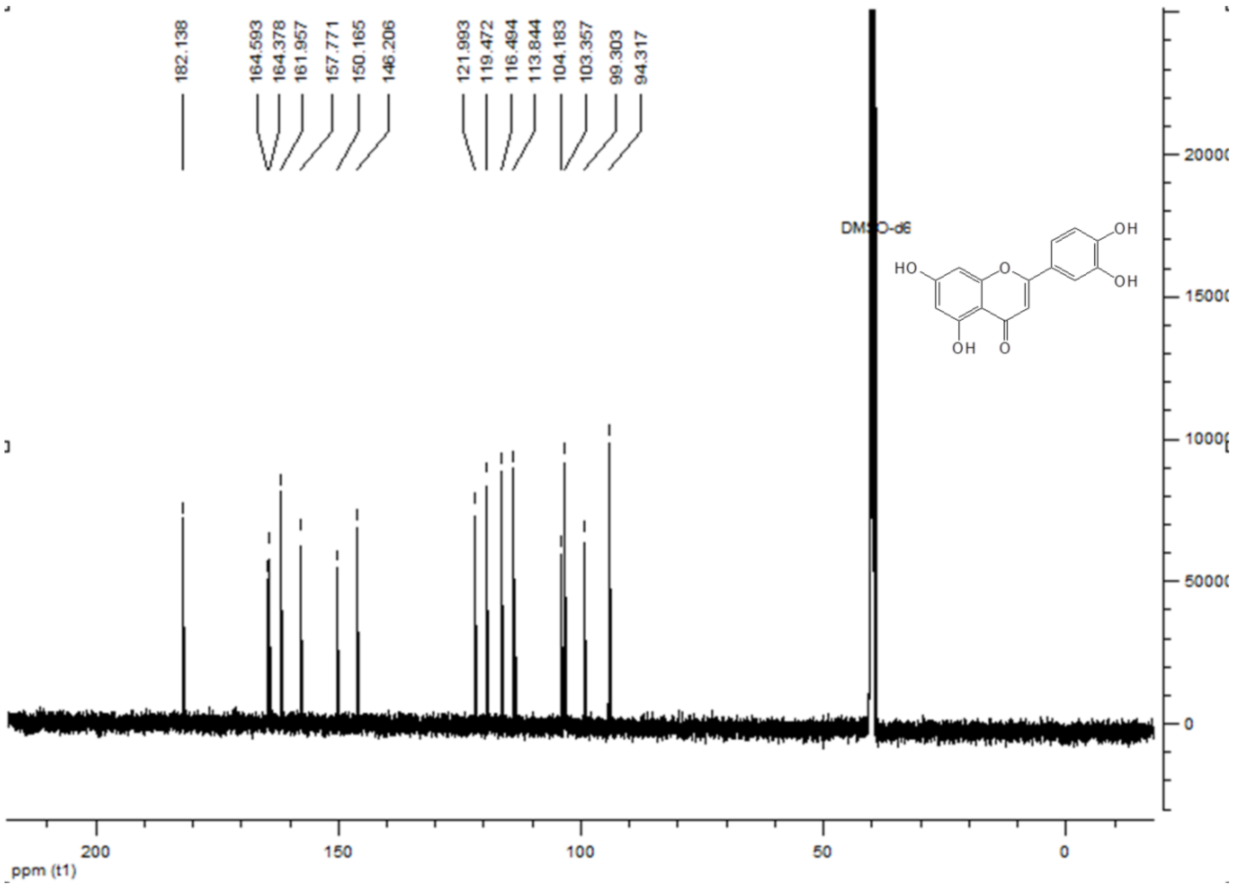

Figure S16. ^{13}C NMR spectrum of compound **8**.
